# Feeding/Eating problems in children: Who does (not) benefit after behavior therapy? A retrospective chart review

**DOI:** 10.3389/fped.2023.1108185

**Published:** 2023-02-28

**Authors:** Eric Dumont, Anita Jansen, Pieter C. Duker, Daniel M. Seys, Nick J. Broers, Sandra Mulkens

**Affiliations:** ^1^ Deparment of Research and Development, SeysCentra, Malden, Netherlands; ^2^ Department of Clinical Psychological Science, Faculty of Psychology and Neuroscience, Maastricht University, Maastricht, Netherlands; ^3^ Department of Psychiatry & Neuropsychology, Faculty of Health, Medicine, and Life Sciences, Maastricht University, Maastricht, Netherlands; ^4^ Department of Methodology & Statistics, Faculty of Psychology and Neuroscience, Maastricht University, Maastricht, Netherlands

**Keywords:** ARFID, behavior therapy, pediatric feeding problems, predictors, prognosis, avoidant/restrictive food intake disorder, treatment

## Abstract

**Background:**

Treating disordered feeding at a young age reduces risks of future feeding problems, but not all children profit equally; can we define predictors of a worse prognosis?

**Objectives:**

In 252 children, with a mean age of 4; 7 years (SD = 3 years; range 5 months to 17; 10 years), who had undergone behavioral day treatment in the past, several variables were investigated, retrieved from initial consultation (t1) and re-assessed at follow-up (t2).

**Method:**

Logistic regressions were carried out with sex, gastro-intestinal problems, refusal of the first nutrition, syndrome/intellectual disability, Down's syndrome, autism spectrum disorder, comorbidity of medical diseases (other than gastro-intestinal problems), restrictive caloric food intake and selective food intake, as the predictor variables from t1, and age-appropriate food intake at t2 as the dependent variable. The potential role of sensory processing problems was reviewed at t2.

**Results:**

About 73% had improved towards an age-appropriate food intake. Sex (boys), syndrome/intellectual disability, and a lack of varied nutritional intake at t1 were predictors of a worse prognosis. We found a small, but significant correlation between current selective eating patterns and general sensory processing problems.

**Conclusion:**

Feeding disordered children, especially boys, with intellectual disabilities or selective eating patterns are at risk for not achieving an age-adequate food intake at a later age, despite behavioral treatment.

## 1. Introduction

Feeding and eating problems are common in young children; they are often characterized by avoidant and/or restrictive eating patterns, and expressed in varying intensity: from picky eating to total food refusal. Problems may be temporary, but some children are at risk for persisting in disordered feeding/eating. The Diagnostic and Statistical Manual of Mental Disorders (DSM-5) (1) describes clear criteria for Avoidant/Restrictive Food Intake Disorder (ARFID), with three representations that drive the avoidant and/or restricted food intake, namely (I) an apparent lack of interest in eating or food; (II) avoidance based on the sensory characteristics of food; and (III) concern about aversive consequences of eating (1–4). Several studies estimated the prevalence of disordered feeding in children. ARFID is estimated to be present in 1.5%–3% of children between 8 and 18 years in the general population (5, 6). In a clinical day treatment setting for eating disorders, however, ARFID seemed to be present in 22.5% of young children and adolescents (7).

The literature indicates multiple factors that may contribute to the development and maintenance of disordered feeding and which could interfere with a successful feeding “career”. In children with intellectual disabilities, cases of disordered feeding as high as 80% are reported (8). Rates increase with greater cognitive impairments and more decline in gross motor function (9–11). Calvert et al. (12) found that half of the children with Down's syndrome ate less than the allowances for food energy and consumed only a limited variety of food and few fruits and vegetables, and Hopman et al. (13) found an increased incidence of texture problems as a result of oral motor delays in a Dutch sample of children with Down's syndrome, compared to typically developing children.

ARFID was reported in children with gastrointestinal symptoms, a history of vomiting/choking, and a comorbid medical condition (6). The odds of suffering from disordered feeding increase by 5 times in children with an autism spectrum disorder (ASD) compared with children without ASD (14). Nadon et al. (15) found an association between general sensory processing problems and eating problems in 65% of the participants (*N *= 95 children) with ASD. These sensory processing problems consisted of hyper- and/or hyposensitivity to tactile stimuli, odor stimuli, texture, color and temperature.

An association between sensory hypersensitivity and picky eating behavior in typically developing children was also found by Nederkoorn and colleagues (16). In this study, 44 children between 4 and 10 years were asked to touch different tactile stimuli with their hands and to taste different foods. Results showed a significant positive correlation between the evaluations of the two modalities, especially in younger children. This suggests that tactile sensitivity might play a role in the acceptance of food.

Regarding treatment, Bourne et al. (17) reviewed diagnostic and treatment options for ARFID in children and found only two (small) RCTs (18, 19). Sharp et al. (18) investigated the effects of a 10-day inpatient behavioral intervention in young children (*N* = 20; age 1–3 years); outcomes were satisfactory. Improvement was observed in all primary outcome measures (bite acceptance, disruptions, and grams consumed during meals) and a high rate of satisfaction was reported at three months follow-up. The other RCT (19) consisted of a double blind, placebo-controlled study, in which 16 children (aged 1.5–3 years) received a behavioral intervention supplemented with or without the drug D-cycloserine, an antibiotic used to treat tuberculosis and applied to enhance exposure-based treatment for anxiety (20, 21). Both groups improved, and an enhanced response was observed within the D-cycloserine group. However, this study included some methodological caveats and results should be considered in this perspective. In addition to these rather small RCTs, most evidence comes from case reports (3, 22–24) and retrospective chart reviews (25, 26). To improve the power of all these smaller studies, Sharp and colleagues (26) conducted a systematic review and meta-analysis of intensive multidisciplinary interventions for pediatric feeding disorder. They found 11 studies involving 593 participants, including nine studies where outcomes were based on retrospective (nonrandomized) chart reviews. All samples involved children with complex medical and/or developmental histories and persistent feeding concerns, similar to ARFID. Medical literature on multidisciplinary treatment of pediatric feeding disorder was reviewed regarding inpatient and outpatient treatment, treatment models and outcome measures. Eight studies involved inpatient treatment, and all programs included psychologists, physicians and nutritionists and in nine studies a speech-language pathologist. In most studies behavioral intervention techniques represented the most common treatment approach. Results indicated that intensive multidisciplinary treatment holds benefits for children with severe feeding difficulties.

In general, populations of children and adolescents with ARFID compared with those with Anorexia Nervosa or Bulimia Nervosa were found to have a greater proportion of boys, although still predominantly girls (6, 7). So far, literature lacks evidence that males respond better or worse to treatment, or relapse earlier, than females.

Despite the promising outcomes of these studies, they do not provide any information on long-term effects or predictors for a worse prognosis. The present study seeks to explore predictors of an adverse future food intake by following-up a large cohort of children who underwent an intense behavioral program which was comparable to that described in the literature. This behavioral treatment program (named “SLIK”) was developed in The Netherlands (27), and entails a twelve-step feeding program based on chaining, differential reinforcement techniques, escape extinction and gradual exposure. In addition to the step-by-step protocolled systematic desensitization procedure, Applied Behavior Analysis techniques are used, which show similarities with the behavioral interventions described in Sharp's (26) systematic review. Five modalities are subject to behavioral change in food acceptance: (1) Texture differentiation (from liquid to solid food); (2) increase in volume and caloric intake; (3) variation in food products (extension and completeness of the diet); (4) temperature differentiation (from cold to warm/hot food-items); (5) socially adapted feeding/eating behavior (e.g., introducing spoon, fork, cup, or moving from only in-session acceptance to implementing the mealtime behavior to social situations with parents, family, and other social settings such as school). In order to properly determine and observe the steps of progress with regard to these modalities, the behavioral therapist is supported by a speech therapist, nutritionist/dietician, a pediatrician and a physical therapist. Each child follows a daily program including four daily treatment sessions. After each step meeting the target behavior criterion, parents are involved to continue the learned step first in the treatment center and then at home under the therapist's supervision. In several case studies (28, 29) and a retrospective analysis (30), SLIK was found to be an effective intervention for achieving a normalized feeding pattern in (young) children with feeding problems, even when they were tube fed.

Although the above studies imply that an intensive behavioral intervention is effective in reducing disordered feeding in young children, clinical practice shows that not all children benefit optimally, and persist in disordered feeding at a later age. Some patients may show a relapse after successful treatment. It is unclear which factors predict who will and who will not achieve an age-appropriate food intake (years) after treatment. Therefore, we aimed to identify predictors from the initial consultation (t1) at a tertiary center for feeding/eating problems that are related to a continuation of disordered feeding at a later age (t2). To this end, we contacted all children who had received an intensive day treatment, several years after finishing this treatment, in order to investigate their present state of feeding/eating and current sensory processing problems. Several variables at t1 were taken from these children's files to investigate their predictive value regarding the present state.

Due to the retrospective nature of this study and its start before ARFID was officially included in the DSM-5, participants received no official ARFID diagnosis as measured by standardized measures. Therefore, we decided to describe these problems as “disordered feeding” and to take “age-appropriate food intake” as the outcome measure. Still, the feeding and eating problems that are treated in our last resort treatment center, have always been identical to what we now call (severe) ARFID.

In this study we investigated the following question: What percentage of children who had received an intensive behavioral day treatment in the past for their disordered feeding/eating patterns did (not) display an age-appropriate food intake, several years post treatment? We hypothesized based on the literature that the following variables might be associated with a worse outcome: (1) an Intellectual Disability (ID) or related syndrome; (2) pre-dysmature birth; (3) Autism Spectrum Disorder (ASD); (4) Down's syndrome; (5) serious gastro-intestinal problems; (6) organic failures/diseases; (7) (male) sex; (8) cultural background (Caucasian vs. non Caucasian); (9) refusal of the first nutrition after birth, and (10) (older) age. We investigated whether any of these variables were related to a present age-(*in*)appropriate food intake. Furthermore, we investigated the relationship of these ten variables with current sensory processing problems by correlating them with the parents' scores on a sensory processing measurement at t2.

## 2. Method

### 2.1. Participants

The parents of all children (*N* = 291) who had entered the intake phase, and obtained treatment between 2009 and 2015, at a specialized treatment center for children with disordered feeding or eating, were invited for participation in this study by letter. The parents of 39 children did not respond or refused participation. The remaining sample consisted of parents of 252 children (109 girls (43.3%) and 143 boys (56.7%)) with a mean age of 4; 7 years (SD = 3 years; range 5 months to 17; 10 years) at initial consultation (t1). During the telephone survey (t2), which took place after - on average - 4; 8 years after t1, their mean age was 9; 3 years (SD = 3; 6 years; range 4; 3–20; 5 years).

### 2.2. Ethical approval

The study was approved by the Ethical Review Committee of the Faculty of Psychology and Neuroscience (ERCPN), Maastricht University (ERCPN_20180222_0490).

### 2.3. Procedure

All parents of the children involved were referred to the specialized treatment center by a pediatrician. At initial consultation, a multidisciplinary team consisting of a pediatrician, dietician, and psychologist examined the child and obtained a history of the child's feeding and several other relevant variables. During the initial consultation (t1), the child's feeding pattern was determined and a treatment advice was provided. The children in the present study all had serious feeding/eating problems and participated in the behavioral day treatment program (4 treatment sessions a day, 5 days a week). The treatment involved a (cognitive) behavioral procedure, named SLIK, which is described in the introduction section. In addition to the behavioral therapists, a dietician, speech therapist and a pediatrician were also involved to monitor each child's treatment steps towards an adequate food intake, oral motor skills, body weight and other health issues.

The treatment had a varying duration, depending on pre-defined (individual) treatment goals taking into account the severity of the feeding/eating problems, child's age, cognitive abilities and underlying problems, and parents' potential. The treatment ended when these goals were achieved. If possible, a more varied diet and a healthy weight were pursued, and the use of medical and/or tube feeding was phased out. The average duration of the inpatient behavioral treatment was 7 months, (range 4–14 months).

At a second time point (t2) which occurred, on average, 4; 8 years after t1, the parents of the children whose treatment had ended in that previous period, and had agreed to participate in the study were approached to take part in a telephone survey, to discuss their child's current feeding/eating status and to take the sensory profile questionnaire.

### 2.4. Design

The study was a retrospective correlational study, with measurements taken at two time points (t1 and t2). We aimed to establish which, if any, variables measured at t1 could successfully predict an age-(*in*)appropriate eating pattern at t2.

#### 2.4.1. T1 measurements

First, all binary variables pertaining to a characteristic were coded “0” if absent and “1” if present (e.g., a child scored “0” on “medical comorbidity” when no comorbid medical problems were present, and “1” when a problem in accordance with the definition of “medical comorbidity” below was mentioned).

(a) *Sex* (i.e., male coded as “0” vs. female coded as “1”).
(b) *Ethnicity* (i.e., Caucasian coded as “0” vs. non-Caucasian coded as “1”).
(c) *Prematurity/dysmaturity* (i.e., prematurity: the child was born before 37 weeks of pregnancy, and dysmaturity: an intrauterine growth restriction which means the child has a birth weight less than 2,500 grams but was born on time. The child can classify on either or both of these conditions. In the first case, it is referred to being born with either of these separately, the latter case is indicated as pre-dysmaturity.
(d) *Medical comorbidities at t1* (specifically, organic failures/diseases *other than* gastro-intestinal problems, such as kidney disease, cardiac problems, metabolic disease).
(e) *Presence of gastrointestinal disease at t1* [e.g., gastro-esophageal reflux disease (GOR), or celiac disease, or delayed gastric emptying, or food allergy].
(f) *Down's Syndrome (DS)*.
(g) *Having a delineated* genetic *syndrome* [i.e., a syndrome with phenotypical eating disturbances other than Down's Syndrome, e.g., Angelman syndrome, Silver Russel syndrome, Noonan syndrome, and/or having an *intellectual disability* with an Intelligence Coefficient (IQ) less than 70].
(h) *Autism Spectrum Disorder (ASD)*.
(i) *Refusal of the first nutrition after birth* (that is, whether the child had refused his nutrition from first bottle or breast immediately after being born).
(j) *Age* at t1.
(k) *Latency* time between t1 and t2.

Next, we reviewed the child feeding performance on a 12-item rating scale derived from the Seyshuizen Food Refusal Questionnaire (SFRQ 31);, which was a standard part of the initial consultation (t1). This scale represents a severity score of food acceptance in terms of texture differentiation, varied food intake and oral-tube ratio, which could range from “1” (i.e., “child refuses any orally presented food”, “requiring tube feeding”) to “12” (“child eats fully orally and accepts food items with a solid texture”). To verify the results of the questionnaire with physical measures, wereviewed the participants' (medical) files during t1 and assessed them on measures (body weight/length, nutritional assessment in response to a food diary, pediatric growth chart observation) which refer to the presence or absence of a “Selective Food Intake” (SFI), (which means a score on either Lack of Varied Nutritional Intake’ (LOVNI), or “Selective Texture Choices” (STC) or both), and secondly the presence or absence of a “Restrictive Caloric Food-Intake (RCFI)”. These were coded “0” when absent and “1” when present. Selective food intake (SFI) indicates (1) a child who repeatedly refused (nearly all) food items from one or more categories from the five basic food groups (of the Dutch equivalent of “MyPlate”) without using any compensation (such as in the case of vegetarian eating) and/or (2) who repeatedly refused food items with a specific texture or bite. The behavior was labeled as a lack of varied nutritional intake (“LOVNI”) when the acceptance per food group was too low, e.g., eating just one or a few bites of one or two types of vegetables or fruits. Avoidance of food items for medical reasons, such as allergies, was excluded. When children repeatedly refused certain texture(s), e.g., only ate grinded or liquid foods, this was labeled as selective texture choices (“STC”). The category restrictive caloric food intake (“RCFI”) was scored positive if the child was (partly) tube–dependent and/or significantly underweight and/or used medical nutrients to provide in its daily quantity of an age-appropriate caloric intake, in accordance with the Dutch Youth Health Care Guidelines in Eating and Feeding Behavior (NCJ: JGZ guidelines, 2013). These binary variables “RCFI”, “SFI”, “LOVNI” & “STC” combined determined whether the child classified as having an age-*appropriate* food intake (code 1), or age-*in*appropriate (code 0) at t1. If the child scored “no” (code 0) on all these binary variables (“SFI”, “LOVNI”, “STC” and “RCFI”) rated at t1, this was ranked as “age-appropriate” (referred to as “AAFI”). If the code on at least one of these variables was “1”, this was ranked as age-*in*appropriate food intake. Logically, all children classified as age-*in*appropriate at t1.

#### 2.4.2. T2 measurements

The aim of the telephone interview (t2) was to collect information about the children's current status to determine the child's food intake, with similar qualifications as at t1, *via* a standardized flow-chart. Besides that, the interviewer rated the child's behavior on the Sensory Profile (32) by questioning the parent(s) on 28 relevant items about sensory processing problems divided over 7 subscales. Ratings were given on a 5-point Likert scale (1 = never, 2 = incidental, 3 = sometimes, 4 = often, 5 = all the time), in the areas of (a) tactile sensitivity, (b) taste-smell sensitivity, (c) movement sensitivity, (d) under responsiveness/seeking sensation, (e) auditory filtering, (f) low energy/weak and (g) visual/auditory sensitivity. Higher scores indicate more sensory processing problems.

The independent interviewer was well experienced in conducting telephone surveys, and she was blind with regard to the child's specific feeding problems and background variables. The survey and the SP rating took 30 min, on average.

Afterwards, it was determined by two researchers, independently, whether the child could be classified as having an age-*in*appropriate food intake (code “0”), or not (code “1”) at t2. In addition to the observed t2 variables, the *latency* time (in years/months) between t1 and t2 was calculated.

### 2.5. Data reduction and analysis

#### 2.5.1. Inter-rater reliability of the assessment of the survey results

To compile a representative group, 7 (t2) surveys corresponding with each year of consultation (between 2009 and 2015), were randomly chosen by an employee who was not involved in the research. Forty-nine surveys (19.4% of all surveys) were randomly selected and independently rated by 2 observers. For every survey, each observer rated the (1) SFRQ scale score (score range 1–12) and the binary variables (2) “RCFI”, (3) “LOVNI”, (4) “STC” and (5) “AAFI” (score 0 or 1) which amounts to a total of 245 (49 × 5) observed variables, each which were compared afterwards on agreement. Inter-rater reliability was determined by calculating Cohen's Kappa.

#### 2.5.2. Statistical analysis

All statistical analyses were performed using SPSS 27. To examine the sample characteristics, we first conducted univariate analyses by calculating frequencies, means and standard deviations of the background variables, and presence and absence of all t1 variables, followed by frequencies and percentages of the dependent variables: restrictive caloric food intake (“RCFI”), selective food intake (“SFI”), lack of varied nutritional intake (“LOVNI”), selective texture choices (“STC”) and age-appropriate food intake (“AAFI”) at t1 and t2. We calculated significance for differences by a paired samples *t*-test and determined the effect sizes using Cohen's *d* for differences between t1 and t2 outcome variables. Then, we computed correlations between all t1 and t2 variables followed by a binary logistic regression to assess whether one or more of the covariates proved predictive of an age (*in*)adequate food intake at t2. Due to the large number of predictors, significance was judged using an α level of 10%. This adjustment of the conventional significance level, which is common in exploratory backwards regression analyses, was chosen to decrease the probability of type II errors. The last step was to compute correlations between the predictor variables as well as the outcome variables RCFI, SFI, LOVNI, STC at t2 with the total score on the “Sensory Profile” at t2 to determine any relation between these variables and sensory processing problems.

## 3. Results

### 3.1. Interrater reliability from the assessment of the survey results

We first determined the inter-rater agreement concerning the assessment of the survey results at t2 of the coded variables. A Kappa of 0.98 was obtained, indicating excellent agreement. Of the observed 245 ratings over the 49 surveys, the two independent raters disagreed about 3 ratings. These ratings were subsequently discussed, to reach agreement.

### 3.2. Description of the sample

The presence of gastro-intestinal diseases at t1 was remarkably high: in 73.4% (185 children). In addition, 97 children (38.5%) were diagnosed with comorbid organic diseases other than gastro-intestinal disease. Fifty-five children (21.8%) were born prematurely and/or dysmaturely. A delineated genetic syndrome (excluding Down's Syndrome) or intellectual disability (IQ < 70) was present in 67 (26.6%) children. DS was seen in 16 children (6.3%), and 34 (13%) of the children were diagnosed with ASD by a previous care provider. The occurrence of comorbid mental and organic involvement are displayed in Table 1.

**Table 1:** Occurrence of comorbid mental and organic involvement within the sample (*N* = 252).

	Sum	Mean (%)
Gastro-intestinal problems (GIT)	185	73.4
Comorbid organic diseases	97	38.5
Syndrome/Intellectual disability (Syndr/ID)	67	26.6
Pre/dysmaturity born	55	21.8
Autism Spectrum Disorder (ASD)	34	13.0
Down's Syndrome (DS)	16	6.3

Restrictive caloric food intake (“RCFI”) was observed in 164 (65%) of the sample, while selective food intake (“SFI”) was seen in 179 (71%). Within the “SFI” group, 39% (98 children) were familiar with a lack of varied nutritional intake (“LOVNI”) and another 47% (119 children) with (also) selective texture choices (“STC”). At t1, none of the 252 children showed an age-appropriate food intake (AAFI). At t2, 27% of the children had *not* acquired an age-appropriate eating pattern. Cohen's *d* showed an effect size of −1,607 which is considered to be a (very) large effect size. The variable “restrictive caloric food intake” (RCFI t1:65%/t2:14%) showed also a great improvement with a Cohen's *d* of 0,995 which is considered to be a large effect size. The variables “lack of varied nutritional intake”, (LOVNI; t1:39%/t2:5%) with a Cohen's *d* of 0,712 and “selective texture choices”, (STC; t1:47%/t2:13%) with a Cohen's *d* of 0,689 show somewhat smaller rates of improvement, but are still considered as medium to large effect-sizes. Overall, selective food intake (SFI; t1:71%/t2: 21%), showed a substantial decrease, with a large effect size (Cohen's *d *= 0.866) but had still the highest presence (21%) at t2 of all variables regarding an age-*in*appropriate food intake. Table 2 displays frequencies, percentages and effect sizes of variables used to classify a child as age-(in)appropriate. The table shows the differences in frequencies and their significance between t1 and t2 in food selectivity' ((a) “LOVNI” and (b) “STC” and (c) “SFI” (total score)), restrictive eating (“RCFI”), and an age adequate food intake (“AAFI”).

**Table 2:** Comparison of frequencies, percentages and effect sizes of dependent variables between t1 and t2 (*N* = 252).

	Frequency t1	Percentage	Frequency t2	Percentage
**Restrictive Caloric Food Intake (RCFI)**				
absence	88	35	216	86
presence	164	65	36	14
Cohen's *d* effect size t1–t2	0.955			
**Selective Food Intake (SFI)**				
absence	73	29	200	79
presence	179	71	52	21
Cohen's *d* effect size t1–t2	0.866			
**Lack of Varied Nutritional Intake (LOVNI)**				
absence	154	61	239	95
presence	98	39	13	5
Cohen's *d* effect size t1–t2	0.712			
**Selective Texture Choices (STC)**				
absence	133	53	220	87
presence	119	47	32	13
Cohen's *d* effect size t1–t2	0.689			
**Age-Appropriate Food Intake (AAFI)**				
absence	252	100	67	27
presence	0	0	185	73
Cohen's *d* effect size t1–t2	−1.607			

It appears from the findings on the SFRQ that feeding skills (related to the need for tube feeding and texture differentiation), in general, improved over time. The mean score at initial consultation (t1) was “6”, as opposed to “11” (range 1–12) at t2. This score suggests that there was, in general, a decrease in (partly) tube feeding dependency, indicating an improvement in oral acceptance and, largely, in texture differentiation. At t1, a score of “1” (“Child refuses any orally presented food, requires tube feeding”) was observed as the most common observation, namely in 67 (27%) of the children, while at t2 this score was observed in just 6 (2%) of the children. An opposite shift was observed with regard to item 12 (“Child eats fully orally and accepts food items with a solid texture”). At t1, score “12” was present in only 58 children (24%), as opposed to 198 children (79%) at t2.

### 3.3. Correlations

We conducted bivariate correlations on all t1 and t2 variables including scale scores of the SFRQ t1 and t2 and the “Sensory Profile” at t2. In Table 3 results are displayed. A weak, but significant negative correlation was found between the child's sex and having a lack of varied nutritional intake (“LOVNI”) at t1 (*r* = −0.335, *p *= 0.01), indicating that boys more often had a lack of varied food intake than girls at initial consultation. Another significant but weak correlation was found between the variable “LOVNI” and “age” at t1 (*r* = 0.307 *p* = 0.01) which means that children with “LOVNI” on t1 were, on average, older than children without “LOVNI”. A third (negative) significant weak correlation was found between “age” at t1 and “gastro-intestinal problems” (*r* = −0.311, *p* = 0.01), indicating that children with these problems were, on average, younger than children without these diseases. Between the two main selective and restrictive eating variables representing an age-*in*appropriate food intake at t1 namely: “SFI” and “RCFI”, a significant weak negative correlation (*r* = −0.413; *p* = 0.000) was found meaning that children with a typical selective eating representation do not automatically display a restrictive caloric food intake and vice versa. Especially the selective food intake (SFI) representation “LOVNI” seems responsible for this weak to moderate correlation (*r* = −.491; *p* = 0.000) relative to “STC” (*r* = 0.076; *p* = 0.236). This suggests that the above found negative correlation between “RCFI” and “SFI” might especially be true for children with a lack of a varied intake and less for children with texture problems. A similar negative but almost negligible correlation (*r* = −.298; *p* = 0.000) was found between these selective eating representations (“STC” and “LOVNI”) at t1 which might indicate that being selective in texture choices does not automatically imply having or developing a lack of varied food intake. Another weak but interesting (negative) correlation between “Syndrome/Intellectual Disability (Syn/ID)” and AAFI at t2 was found (*r* = −0.187, *p* = 0.003) which indicates that children without a syndrome and/or intellectual disability more often achieved an age-appropriate food intake at t2 than children with a syndrome and/or ID. However, given that the strength of significance is below 0.3, this correlation is negligible.

**Table 3:** Correlations of all t1 variables with all t2 outcome measures.

	SP-totalscore	Sex	Etnicity	Pre/dysmature	Refusal first nutrition	GIP	Comorbidity	Syndr/ID	Down Syndrome	ASD	SFI (t1)	SFI (t2)	RCFI (t1)	RCFI (t2)	AAFI (t2)	SFRQ (t1)	SFRQ (t2)	LOVNI (t1)	STC (t1)	LOVNI (t2)	STC (t2)	Latency t1-t2	Age t1
SP-totalscore	Pearson Correlation	1																					
	Sig. (2-tailed)																						
	N	230																					
Sex	Pearson Correlation	−0.123	1																				
	Sig. (2-tailed)	0.064																					
	N	230	252																				
Etnicity	Pearson Correlation	0.107	−0.098	1																			
	Sig. (2-tailed)	0.107	0.122																				
	N	230	252	252																			
Pre/dysmature	Pearson Correlation	0.129	0.023	−0.042	1																		
	Sig. (2-tailed)	0.051	0.711	0.510																			
	N	230	252	252	252																		
Refusal first nutrition	Pearson Correlation	.185**	0.012	−0.002	.304**	1																	
	Sig. (2-tailed)	0.005	0.854	0.974	0.000																		
	N	230	252	252	252	252																	
GIP	Pearson Correlation	0.049	0.054	0.070	0.014	.154*	1																
	Sig. (2-tailed)	0.462	0.393	0.270	0.831	0.015																	
	N	230	252	252	252	252	252																
Cormorbidity	Pearson Correlation	0.049	−0.032	−0.010	0.076	.162*	0.051	1															
	Sig. (2-tailed)	0.459	0.611	0.878	0.232	0.010	0.416																
	N	230	252	252	252	252	252	252															
Syndr/ID	Pearson Correlation	0.101	0.091	0.066	0.052	.155*	0.098	.170**	1														
	Sig. (2-tailed)	0.127	0.150	0.295	0.414	0.014	0.121	0.007															
	N	230	252	252	252	252	252	252	252														
Down Syndrome	Pearson Correlation	0.051	−0.030	0.049	−0.059	0.073	0.009	0.028	.433**	1													
	Sig. (2-tailed)	0.444	0.633	0.439	0.353	0.251	0.883	0.657	0.000														
	N	230	252	252	252	252	252	252	252	252													
ASD	Pearson Correlation	0.108	−.125*	0.112	−0.006	0.000	−.166**	−.138*	0.006	−0.101	1												
	Sig. (2-tailed)	0.101	0.047	0.076	0.927	0.996	0.008	0.029	0.924	0.109													
	N	230	252	252	252	252	252	252	252	252	252												
SFI (t1)	Pearson Correlation	0.074	−.202**	0.050	−0.023	0.053	−0.107	−0.052	−0.071	0.023	.170**	1											
	Sig. (2-tailed)	0.263	0.001	0.431	0.721	0.402	0.090	0.410	0.261	0.719	0.007												
	N	230	252	252	252	252	252	252	252	252	252	252											
SFI (t2)	Pearson Correlation	.267**	−.148*	0.077	−0.008	.137*	0.041	−0.041	0.115	0.028	0.093	0.088	1										
	Sig. (2-tailed)	0.000	0.019	0.222	0.896	0.029	0.522	0.521	0.069	0.657	0.142	0.164											
	N	230	252	252	252	252	252	252	252	252	252	252	252										
RCFI (t1)	Pearson Correlation	0.091	.253**	0.020	0.105	.124*	.200**	0.118	0.083	−0.082	−.283**	−.413**	0.003	1									
	Sig. (2-tailed)	0.171	0.000	0.752	0.097	0.049	0.001	0.062	0.190	0.193	0.000	0.000	0.959										
	N	230	252	252	252	252	252	252	252	252	252	252	252	252									
RCFI (t2)	Pearson Correlation	0.030	0.056	−0.031	0.031	0.039	0.066	0.050	.191**	−0.106	−0.024	−.139*	.240**	.204**	1								
	Sig. (2-tailed)	0.649	0.380	0.628	0.620	0.534	0.297	0.430	0.002	0.092	0.704	0.027	0.000	0.001									
	N	230	252	252	252	252	252	252	252	252	252	252	252	252	252								
AAFI (t2)	Pearson Correlation	−0.127	0.072	−0.032	−0.030	−0.101	−0.098	0.015	−.187**	0.009	−0.033	0.051	−.670**	−0.064	−.627**	1							
	Sig. (2-tailed)	0.054	0.254	0.610	0.636	0.110	0.121	0.818	0.003	0.883	0.606	0.417	0.000	0.312	0.000								
	N	230	252	252	252	252	252	252	252	252	252	252	252	252	252	252							
SFRQ (t1)	Pearson Correlation	−.140*	−.292**	−0.080	−.130*	−.199**	−.237**	−.189**	−.233**	−0.034	.220**	.323**	−0.067	−.785**	−.235**	.144*	1						
	Sig. (2-tailed)	0.034	0.000	0.204	0.039	0.002	0.000	0.003	0.000	0.593	0.000	0.000	0.291	0.000	0.000	0.023							
	N	230	252	252	252	252	252	252	252	252	252	252	252	252	252	252	252						
SFRQ (t2)	Pearson Correlation	−0.120	−0.053	−0.048	−0.035	−0.085	−.144*	−0.089	−.270**	0.044	0.028	0.067	−.418**	−.210**	−.836**	.711**	.316**	1					
	Sig. (2−tailed)	0.069	0.402	0.445	0.579	0.178	0.022	0.158	0.000	0.487	0.654	0.286	0.000	0.001	0.000	0.000	0.000						
	N	230	252	252	252	252	252	252	252	252	252	252	252	252	252	252	252	252					
LOVNI (t1)	Pearson Correlation	−0.049	−.335**	−0.043	−.126*	−.125*	−0.110	−.146*	−.241**	−0.108	.245**	.366**	0.076	−.491**	−0.116	0.001	.580**	.149*	1				
	Sig. (2-tailed)	0.459	0.000	0.499	0.046	0.047	0.083	0.020	0.000	0.088	0.000	0.000	0.229	0.000	0.065	0.987	0.000	0.018					
	N	230	252	252	252	252	252	252	252	252	252	252	252	252	252	252	252	252	252				
STC (t1)	Pearson Correlation	.165*	0.089	0.061	.154*	.265**	0.047	0.085	.150*	0.080	−0.014	.429**	.186**	0.076	0.000	−0.060	−.293**	−.126*	−.298**	1			
	Sig. (2-tailed)	0.012	0.160	0.334	0.014	0.000	0.453	0.179	0.017	0.207	0.828	0.000	0.003	0.230	1.000	0.339	0.000	0.046	0.000				
	N	230	252	252	252	252	252	252	252	252	252	252	252	252	252	252	252	252	252	252			
LOVNI (t2)	Pearson Correlation	0.086	−.131*	−0.067	−0.036	−0.019	−0.022	−0.037	−0.059	−0.061	0.016	0.109	.457**	−.130*	0.110	−.388**	0.113	−0.121	.292**	−0.113	1		
	Sig. (2-tailed)	0.195	0.037	0.292	0.566	0.766	0.727	0.559	0.350	0.337	0.803	0.083	0.000	0.039	0.082	0.000	0.074	0.056	0.000	0.074			
	N	230	252	252	252	252	252	252	252	252	252	252	252	252	252	252	252	252	252	252	252		
STC (t2)	Pearson Correlation	.200**	0.004	0.117	0.000	.179**	0.095	0.017	.229**	0.096	0.029	0.086	.689**	0.104	.321**	−.607**	−.240**	−.585**	−.133*	.332**	0.019	1	
	Sig. (2-tailed)	0.002	0.952	0.064	0.994	0.004	0.134	0.792	0.000	0.128	0.651	0.174	0.000	0.098	0.000	0.000	0.000	0.000	0.035	0.000	0.766		
	N	230	252	252	252	252	252	252	252	252	252	252	252	252	252	252	252	252	252	252	252	252	
Latency t1–t2	Pearson Correlation	−0.069	.150*	.172**	−0.030	0.085	.128*	−0.004	0.114	.206**	−0.095	−.205**	−0.031	.191**	0.063	−0.024	−.358**	−0.027	−.318**	0.019	−0.110	0.070	1
	Sig. (2-tailed)	0.299	0.018	0.007	0.640	0.183	0.044	0.949	0.071	0.001	0.135	0.001	0.630	0.002	0.321	0.710	0.000	0.672	0.000	0.769	0.083	0.274	
	N	228	249	249	249	249	249	249	249	249	249	249	249	249	249	249	249	249	249	249	249	249	249

Among the t2 variables, significant but weak correlations were observed between the general outcome scale score of the “SP” and the variables “STC” and “SFI”, that could indicate the possible expected relation between sensory processing problems and selective texture choices (STC: *r* = 0.200, *p* = 0.002) as well as sensory processing problems and selective food intake (SFI: *r* = 0.267, *p* = 0.000). However, also these relatively low associations should be interpreted with caution.

The SFRQ-scale score at t2 showed moderate to strong levels of correlation with the disordered eating outcome measures AAFI-t2 (*r* = 0.711, *p* = 0.000), RCFI-t2 (*r* = −0.836, *p* = 0.000), STC-t2 (*r* = −0.585, *p* = 0.000), and SFI-t2 (*r* = −0.418, *p *= 0.000). These correlations were expected and indicate that the SFRQ is an adequate measure to determine age-appropriate food intake.

### 3.4. Predicting age-appropriate food intake at t2

We used a logistic regression model to examine the predictive value of all t1 variables related to the outcome measure age-appropriate food intake at t2. An age-appropriate food intake implies no significant disturbances in the necessary caloric food intake, a varied nutritional food intake and a presentation of all textures in daily meals. In Table 4, the predictors are summarized with relevant odds ratios (OR) and associated confidence intervals (C.I.). Four variables turned out to be significant predictors. The variable “Sex” showed an OR of 1.804 (*p* = 0.077) and appeared to be significant. This implies that boys more often are at risk for *not* achieving an age-appropriate food intake at a later age than girls. Second, “Syndrome/Intellectual Disability” had an OR of 0.424 (*p* = 0.011) which indicates that children with a syndrome and/or intellectual disability are more at risk for *not* achieving age-appropriate food intake than children without. A third significant predictor was found in “LOVNI” with an OR of 0.474 (*p *= 0.081), meaning that a less varied nutritional intake at a younger age predicts age-inappropriate food intake at t2. A fourth predictor was a lower score at the SFRQ-scale at t1 with an OR of 1.151 (*p* = 0.048); Table 4 lists the results for the logistic regression analysis. A preliminary check revealed no significant collinearity between predictors.

**Table 4:** Logistic regression model of all t1 variables in the equation.

	LogOdds	Wald (df = 1)	Sig. (*p*)	Odds Ratio	95% C.I.(OR)	
					Lower	Upper
**Sex**	.590	3.134	.077	1.804	.939	3.465
**GIP**	−.317	.712	.399	.729	.349	1.521
**Comorbidity**	.345	1.160	.282	1.413	.753	2.649
**ASD**	−,217	.221	.638	.805	.325	1.991
**Syndrome/ID**	−.859	6.445	.011	.424	.218	.822
**SFRQ (t1)**	.141	3.906	.048	1.151	1.001	1.324
**RCFI (t1)**	.470	.632	.426	1.600	.502	5.100
**SFI (t1)**	.575	1.569	.210	1.777	.723	4.368
**LOVNI (t1)**	−.746	3.054	.081	.474	.206	1.095
**STC (t1)**	−.368	.821	.365	.692	.313	1.534
Constant	.074	.006	.940	1.077		

AAFI, age-appropriate food intake; GIP, gastro-intestinal problems; ID, intellectual disability; ASD, autism spectrum disorder; SFRQ, seyshuizen food refusal questionnaire; RSFI, restrictive caloric food intake; SFI, selective food intake; LOVNI, lack of varied nutritional intake; STC, selective texture choices; OR, odds ratio; df, degree of freedom; C.I., confidence interval.

As displayed in Table 4, some of the effects in the regression model are not significant, and, thus, unjustly corrected for overlap. Therefore, we added an extra Table 5 to show the significant effects corrected only for the relevant effect between the predictors.

**Table 5:** Logistic regression model after sequential deletion of non-significant predictors (*α* = 0.10).

	LogOdds	Wald (df = 1)	Sig. (*p*)	Odds Ratio	95% C.I.(OR)	
					Lower	Upper
Sex	.535	2.663	.103	1.707	.898	3.245
Syndrome/ID	−.889	7.419	.006	.411	.217	.779
SFRQ (t1)	.117	7.059	.008	1.124	1.031	1.225
LOVNI (t1)	−.637	2.619	.106	.529	.245	1.144
Constant	.606	.352	.085	1.833		

AAFI, age-appropriate food intake; Syndrome/ID, syndrome and/or intellectual disabilities; SFRQ, seyshuizen food refusal questionnaire; LOVNI, lack of varied nutritional intake; t1, timepoint 1; df, degree of freedom; C.I., confidence interval; OR, odds ratio.

### 3.5. Correlations with the sensory profile (SP)

To test the hypothesis that sensory processing problems might influence reaching an age-appropriate food intake, we calculated correlations between SP total scores and all variables at t1 and t2. Unexpectedly, we found only weak positive, though significant correlations of SP with selective eating at t2: SFI (*r* = 0.267, *p* = 0.000) and STC (*r* = 0.200, *p* = 0.002), indicating that children with selective eating patterns and selective texture choices scored higher on the SP than children without these eating patterns. Further, a weak but significant negative correlation was found between SP and SFRQ-t1 (*r* = −0.140; *p* = 0.0340), meaning that lower SFRQ scores at t1 were associated with higher SP scores at t2; however, due to their low strength, these could be interpreted as negligible. Unexpectedly, there was no significant association between autism spectrum disorder (ASD) and the “Sensory Profile” score (*r* = 0.108, *p* = 0.101) at t2.

## 4. Discussion

The aim of this study was to identify predictors of current feeding/eating disorders in children several years after an intensive behavioral intervention. Of 252 children, 73% achieved an-age adequate food intake at t2, which was - on average - 4; 8 years after t1. In the remaining 27% of the children, calculated effect-sizes showed a more or less improved food intake but these children still met increased rates on one or more of the restrictive and/or selective outcome measures at t2. We hypothesized that a set of ten variables might be of influence regarding *not* achieving an age-appropriate food-intake at a later age, despite intensive behavioral treatment. Two of those determinants indeed predicted not developing an age-appropriate food intake (after treatment) at a later age (t2), namely (1) having a syndrome and/or an intellectual disability (excluding Down's syndrome), and (2) being a boy. “Sex” was found to be the variable with the highest odds ratio (*p* = 1.804, *p* = 0.077) which means that boys are almost twice as much more likely to be at risk as girls. Despite boys in general being somewhat underrepresented in ARFID population (6, 7), the present study shows that they have a worse prognosis after treatment, then girls. Though with the note that in this sample both were almost equally represented.

In addition, we investigated whether restrictive (RCFI) and selective eating measures (SFI, STC, LOVNI), and the SFRQ-scale score at t1, predicted an age-(*in*)appropriate food intake (AAFI) at t2. Regarding the eating measures the children with a lack of a varied nutritional intake (LOVNI) at t1 were predictive for *not* achieving an age adequate at t2. Also, the Seyshuizen Food Refusal Questionnaire (SFRQ)-score at t1 turned out to be predictive, indicating that a lower score at t1 was predictive for *not* achieving an age-appropriate food-intake at t2, which means that children who are fully tube fed seems to be more at risk. Concerning the Sensory Profile (SP) total score, we only found several weak, but significant correlations, all below a size of *r *= 0.3. Given that the relationship between sensory processing problems and eating difficulties is observed more often in the literature (33), we expected a stronger association. We also expected a significant correlation between ASD and the SP total score at t2, since in the literature it was found that these children can exhibit high levels of sensory processing problems (SPP), (34, 15). However, in these studies no behavioral treatment was provided. Peterson and colleagues (35) tested the effectiveness of a behavioral intervention in children with ASD and sensory processing related eating problems. The behavioral intervention turned out be very effective in reducing these eating problems. We can only hypothesize that the applied behavioral intervention, which included gradual exposure (and systematic desensitization) might also have caused reduction of sensory processing problems, and that having ASD is not an additional obstacle. But that might also be the case related to remaining determinants defined in this research. In general, no significant relation was found between SPP and remaining in an age-*in*appropriate food intake (at t2) when behavioral treatment had preceded.

Our findings from this retrospective chart study, with regard to the level of the (long term) follow-up effectiveness post behavioral treatment intervention, are in line with the levels found in Sharp's studies (18, 19, 26) on behavioral treatment of pediatric feeding disorders. In Sharp's (26) meta-analysis, including 11 studies, an effect of behavioral treatment procedures was found of 71%–80%. The determinants for problematic feeding patterns that are generally found in the literature, like ASD (14), gastro-intestinal issues (6), being tube fed at a young age (36, 37), and suffering from Down's syndrome (38) turned out to be *not* significantly predictive for not achieving a normalized eating pattern at a later age after behavioral treatment. Thus, behavioral treatment seems to be indeed valuable for children with these issues. Perhaps remarkable was that suffering from Down's Syndrome, which in all cases was associated with intellectual disability, appears to have a better prognosis after treatment, in contrast to the other syndromic pictures with ID. The choice to study this group separately was motivated by our clinical experience that these children are generally well treatable.

In children with a syndrome and/or severe intellectual disability, physical and neurological comorbidities are more common than in typically developing children. This may also explain why they are less able to meet the criteria of an age-appropriate food intake (AAFI) as stated in this study. Afterwards we can discuss whether this outcome measure is relevant for this group. Thus, feeding intervention may significantly improve intake and achieve developmentally appropriate targets, but falls short of age expectations. However, we also see children with these limitations who do meet this criterion.

Having a lack of varied nutritional intake (t1), which also was predictive for not developing an AAFI, could possibly be a better subject of prevention. Confronting children in early stages of life with more varied foods, textures or tastes, if only by systematic touching and tasting, might prevent (increased) eating problems later in life (16).

Limitations of the study concern the use of binary variables instead of Likert type scales which might show more variability. Because of the available chart review data, we chose for these types of variables, which is also inherent to this type of research. In addition to the observed binary variables from the chart reviews, we found supported measures on categorical variables for disordered feeding patterns in the SFRQ and the SP. The SFRQ showed good validity and reliability for the Dutch population (31) and only a minimum of structured valid instruments was available at that time. Another limitation was the heterogeneity of the sample which makes generalization more challenging. We applied a collinearity check to our data to determine a possible problematic overlap between variables regarding typical sample characteristics. No serious collinearity was indicated. We should therefore consider the results in relation to clinical practice research, which we believe, seen our sample size, is valuable. Another limitation concerned the lack of valid ARFID-measurements at the time of data collection during t1. Moreover, the ARFID DSM-5 classification, including its profiles, was not even available at that time. Therefore, the authors could not elaborate on the potential ARFID profiles of the participants. However, we can clearly observe that the clinical manifestations seen in our participants correspond with the present, clinical picture of ARFID. Nevertheless, based on the available file data, the authors chose not to retrospectively assign an ARFID diagnosis, nor any of the profiles as the information about this. They felt this would be unfair as the information was unequally divided over the participant's files (i.e., for some children, this information was present and could have been used, whereas in others, it was unknown). A last limitation involves the comorbidity of psychological, psychiatric and mental issues and its relationship to the eating and feeding problems similar to ARFID. Results from this study show that only children with an intellectual disability might be at risk for a reduced treatment outcome or relapse. Besides ASD and gastrointestinal- and/or other medical diseases, other psychiatric features that might be involved, were not identified as risk factors prior to the study and were, thus, not included. This was partly due to the fact that given the size of the sample, a choice had to be made for a limited number of potential risk factors. For example, it would also have been interesting and valuable to include the role of child–parent interaction as a possible risk factor, as described by Maestro and colleagues (39). However, due to the retrospective nature of the study design, there was insufficient structured file information on this aspect among the majority of the participants, making it impossible to draw retrospective conclusions about this.

The results of this study show a substantial improvement of feeding/eating problems in the long run and we can only substantiate that behavioral treatment may serve as a serious contribution to solving feeding/eating problems in children. We base this assumption also on another study by Dumont and colleagues (40). In a similarly applied retrospective study conducted among a sample of 236 children, of a similar age range and comparable severity, *who refrained from behavioral treatment*, they found that after an average follow-up period of 6; 3 years, only 37% of the participants had developed an age-appropriate eating pattern and 63% still had a restrictive and or selective eating pattern.

In contrast, in this study we found in 73% of the children an age-appropriate food intake, still years after a behavioral intervention; a selection of the sample did not benefit optimally, being at risk for a disordered eating pattern, as with ARFID may be the case. Having a selective eating pattern at a young age, suffering from a syndrome with an intellectual disability and being a boy were found predictive for *not* achieving an age-appropriate food intake. Now that we know more about potential risk factors, we can look more specifically at targeted interventions for these children.

## Data Availability

The datasets presented in this study can be found in online repositories. The names of the repository/repositories and accession number(s) can be found below: dataverse.nl.
